# Yaws

**DOI:** 10.1177/0956462414549036

**Published:** 2014-09-04

**Authors:** Michael Marks, Dornubari Lebari, Anthony W Solomon, Stephen P Higgins

**Affiliations:** 1 Clinical Research Department, Faculty of Infectious and Tropical Diseases, London School of Hygiene and Tropical Medicine, London, UK; 2 The Hospital for Tropical Diseases, Mortimer Market Centre, Mortimer Market, London, UK; 3 Department of Sexual Health and HIV, North Manchester General Hospital, Manchester, UK

**Keywords:** Syphilis, Yaws, *Treponema pallidum pertenue*, non-venereal endemic syphilis, neglected tropical diseases

## Abstract

Yaws is a non-venereal endemic treponemal infection caused by *Treponema pallidum* sub-species *pertenue,* a spirochaete bacterium closely related to * Treponema pallidum ssp. pallidum*, the agent of venereal syphilis. Yaws is a chronic, relapsing disease predominantly affecting children living in certain tropical regions. It spreads by skin-to-skin contact and, like syphilis, occurs in distinct clinical stages. It causes lesions of the skin, mucous membranes and bones which, without treatment, can become chronic and destructive. * Treponema pallidum ssp. pertenue*, like its sexually-transmitted counterpart, is exquisitely sensitive to penicillin. Infection with yaws or syphilis results in reactive treponemal serology and there is no widely available test to distinguish between these infections. Thus, migration of people from yaws-endemic areas to developed countries may present clinicians with diagnostic dilemmas. We review the epidemiology, clinical presentation and treatment of yaws.

## Yaws

Yaws is a non-venereal endemic treponemal infection caused by *Treponema pallidum* sub-species *pertenue*,^1^ a bacterium closely related to * Treponema pallidum ssp. pallidum*, the agent of venereal syphilis. Yaws predominantly affects children living in tropical regions of the world. It causes lesions of the skin, mucous membranes and bones which, without treatment, can become chronic and destructive. There is no widely available test to distinguish yaws from syphilis. Thus, migration of people from yaws-endemic areas to developed countries may present clinicians with diagnostic dilemmas. The other endemic treponemal infections are bejel (endemic syphilis) caused by *Treponema pallidum ssp. endemicum* and pinta caused by *Treponema carateum.*

## Epidemiology

Yaws is currently thought to be endemic in at least 12 countries^2,3^ (Table 1). The number of notified yaws cases is almost certainly an underestimate of true disease incidence. Yaws primarily affects children living in poor, densely-populated rural areas. The concentration of yaws in warm, humid climates is thought to be explained by the sensitivity of *T p pertenue* to relative cool and dryness and may explain why skin lesions are seen more often in the rainy season.^4^

**Table 1.** Countries in which yaws is currently endemic.

Country	Local name
Benin	Not Known
Cameroon	Not Known
Central African Republic	Not Known
Democratic Republic of the Congo	Not Known
Congo	Not Known
Côte d’Ivoire*	Goundou
Ghana	Gyator
Togo	Gbodo, Gbodokui,
Indonesia	Frambusia
Timor-Leste	Not Known
Papua New Guinea	Not Known
Solomon Islands	Yaws
Vanuatu	• 50 vatu soa • bigfella soa

*Endemic status unclear.

In the 1950s it was estimated that 50 million people were infected with yaws. The World Health Organization (WHO) tried to eliminate the disease through a mass treatment campaign using benzylpenicillin.^2,5^ Consequently, the number of infections worldwide dropped significantly but yaws then fell off the public health agenda. The next 30 years saw a resurgence of cases and the disease is, again, a public health problem in Africa,^6^ South-East Asia, the Pacific^7,8^ and South America.^9^ The WHO estimates that 2.5 million individuals may currently be infected.^2^ A failure to identify contacts of infected individuals, inadequate treatment of latent yaws as well as a failure to integrate control efforts into primary health care are thought to have led to the eventual failure of the WHO elimination strategy.^5^

## Transmission

Bacteria from infectious lesions enter via a breach in the skin. Lesions of early yaws are most infectious as they carry a higher bacterial load, whilst late yaws lesions are not infectious. It is estimated that infectivity lasts for 12–18 months after primary infection^1^ but relapsing disease can extend this period (see ‘latency’ below). It has been postulated that infection might be spread by flies^10^ but there is no evidence to support this mode of transmission in humans. Transplacental spread of *T p pertenue* is said not to occur, but this view is disputed.^11^

## Bacteriology

*T p pertenue* is a Gram-negative spirochaete which cannot be cultured *in vitro.*^1^ Five strains have been cultured in rabbits and golden hamsters.^12^ The organism is closely related to *T p pallidum* with a genome that differs by approximately 0.2%. These differences are restricted to a small number of genes including *tpr* and *TP0136.* The role of these genes is uncertain but they have been implicated in pathogenesis.^12^ The phylogenetic relationship of yaws and syphilis remains unclear and there is evidence that recombination between the two organisms can occur.^13^

## Clinical presentation

The clinical presentation of yaws bears similarities to that of syphilis (Table 2). Like syphilis, yaws can be staged as early (primary and secondary) and late, or tertiary. Though clinically useful, this classification is artificial and patients may present with a mixture of clinical signs.

**Table 2.** Comparison of clinical features and timing of yaws and syphilis.

	Syphilis		Yaws	
Primary	Incubation	9–90 days	Incubation	10–90 days
	Morphology	Chancre. Usually solitary, often multiple. Non-tender. Scarring very unusual.	Morphology	Mother yaw. Usually solitary. Non-tender. Scarring usual.
	Site	Ano-genital	Site	Legs, ankles
Secondary	Incubation	Weeks-24 months	Incubation	Weeks-24 months
	Clinical presentation	Skin rash Lymphadenopathy Mucosal lesions	Clinical presentation	Arthralgia Malaise Skin lesions Polyosteitis of fingers, feet or long bones
Latency	Yes		Yes	
Infectious relapses	Commonest within the first two years, rarely thereafter		Up to 5 years, Rarely up to 10 years.	
Tertiary	Clinical		Clinical	
	Cardiovascular (10%)	Decades	??Cardiovascular	5 + years
	Neurosyphilis (10%)	Weeks (meningitis, cranial neuritis) Decades: tabes, GPI	??Neuroyaws	
	Gummata	10–15 years	Gummatous nodules. Scarring, contractures. Gangosa. Tibial bowing. Goundou	
Congenital infection	Yes		No evidence	

## Primary yaws

**Figure 1.**
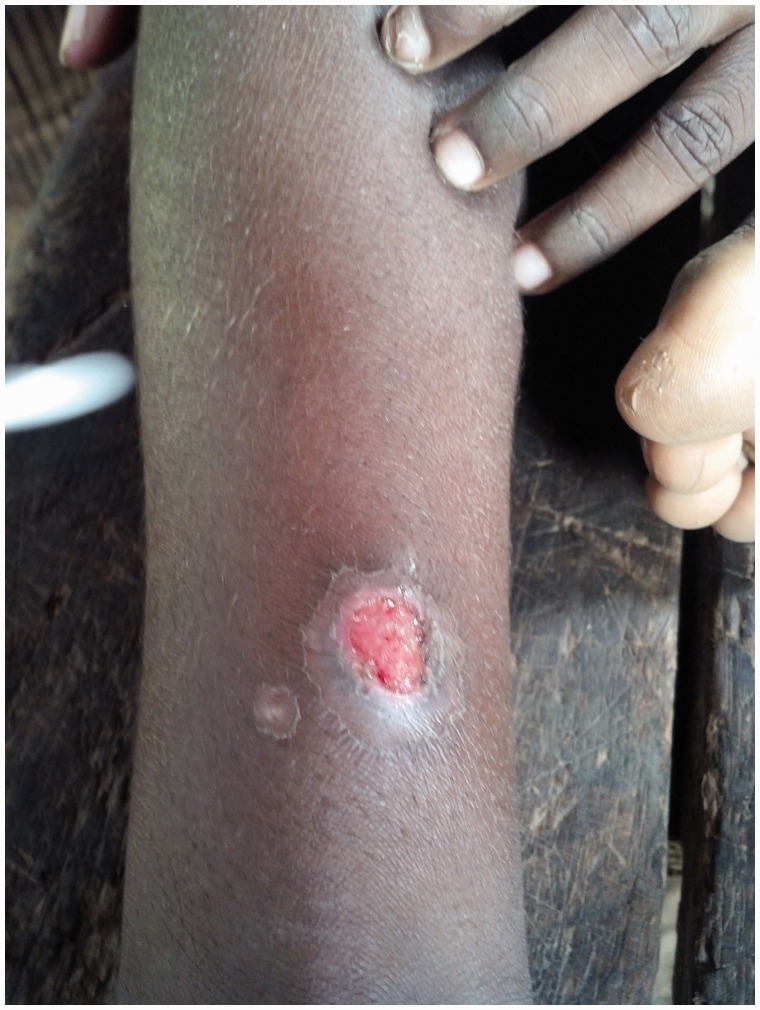
Ulcer of primary yaws. Copyright Michael Marks.

**Figure 2.**
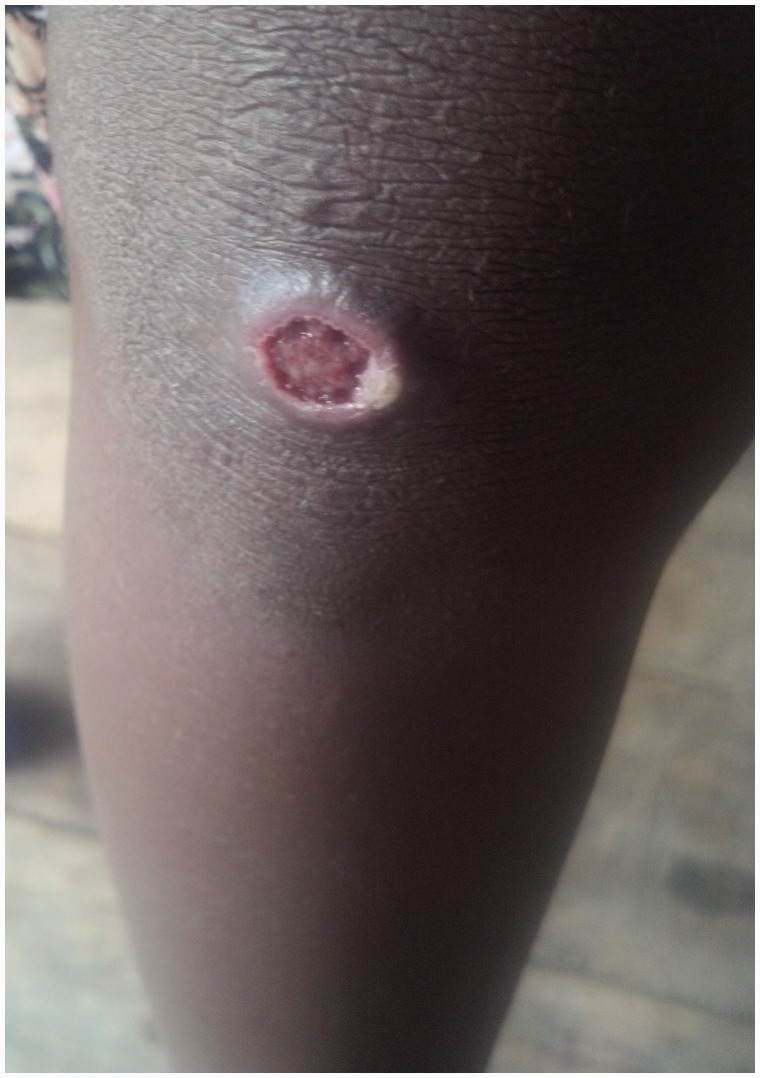
Ulcer of primary yaws. Copyright Michael Marks.

**Figure 3.**
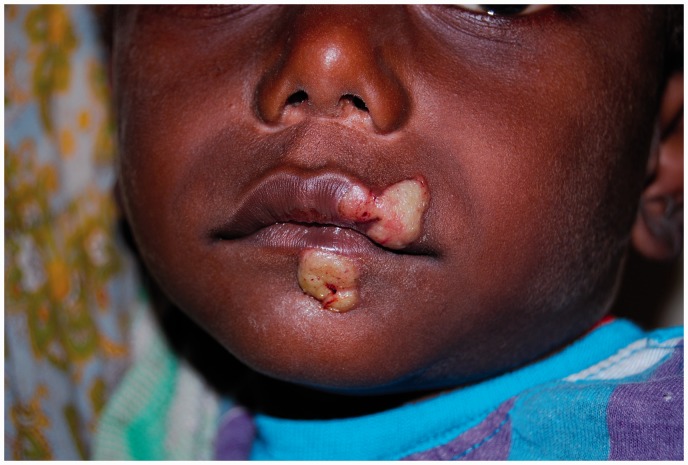
Papilloma of primary yaws. Copyright Oriol Mitjà.

A papule appears at the inoculation site after about 21 days (range 9–90).^1,10^ This ‘Mother Yaw’ may evolve either into an exudative papilloma, 2–5 cm in size or degenerate to form a single, non-tender ulcer (Figures 1–3) covered by a yellow crust. The legs and ankles are the commonest sites affected, but lesions may occur on the face, buttocks, arms or hands.^14^ ‘Split-papules’ may occur at the angle of the mouth.^1^ Regional lymphadenopathy is common. In contrast to syphilis, genital lesions are rare. Primary lesions are indolent and take 3–6 months to heal, more often leaving a pigmented scar.^15^ As in syphilis,^16^ the primary lesion is still present when signs of secondary yaws develop in about 9–15% of patients.^17^

## Secondary yaws

Haematogenous and lymphatic spread of treponemes produces secondary lesions, most commonly one to two months (but up to 24 months) after the primary lesion. General malaise and lymphadenopathy may occur. The most florid manifestations of secondary yaws occur in skin and bone.^14^

### Skin

**Figure 4.**
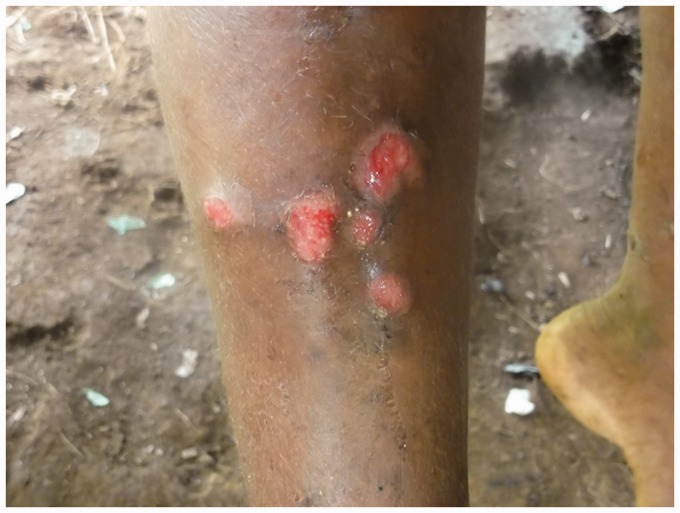
Secondary yaws: multiple small ulcerative lesions. Copyright Michael Marks.

**Figure 5.**
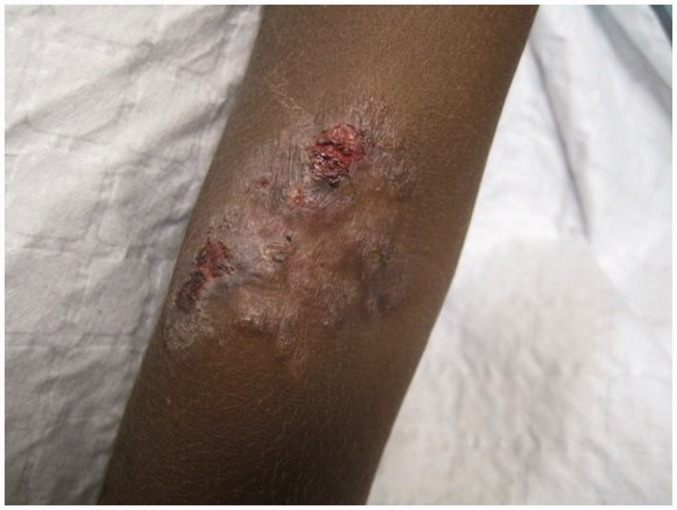
Secondary yaws: maculo-papular lesions with scaling. Copyright Oriol Mitjà.

The rash begins as pinhead-size papules, which develop a pustular or crusted appearance and may persist for weeks. If the crust is removed a raspberry-like appearance may be revealed. Sometimes papules enlarge and coalesce into cauliflower-like lesions, most frequently on the face, trunk, genitalia and buttocks. Scaly macules may be seen (Figures 4 and 5). Lesions in warm, moist areas may resemble condylomata lata of syphilis.

The skin lesions of early yaws are often itchy and the Koebner phenomenon has been observed. Mixed papular and macular lesions are often seen in individual patients. Secondary skin lesions may heal even without treatment, with or without scarring.

Squamous macular or plantar yaws can resemble secondary syphilis.^1^ Lesions on the soles of the feet may become hyperkeratotic, cracked, discoloured or secondarily infected. This can result in pain and a crab-like gait.^18^ Mucous membrane involvement, most commonly nasal, was reported in less than 0.5% of cases in American Samoa.^19^

There is some evidence that the manifestations of yaws in the modern era are less florid than previously reported. It has been postulated that use of penicillins to treat other conditions may be responsible for this. The differential diagnosis of yaws lesions is wide and includes syphilis, leishmaniasis, leprosy and Buruli ulcer, as well as non-infectious causes. Discussion with a physician with expertise in tropical medicine is recommended as the differential diagnosis and choice of investigations will vary depending on the patient’s country of origin.

### Bones

**Figure 6.**
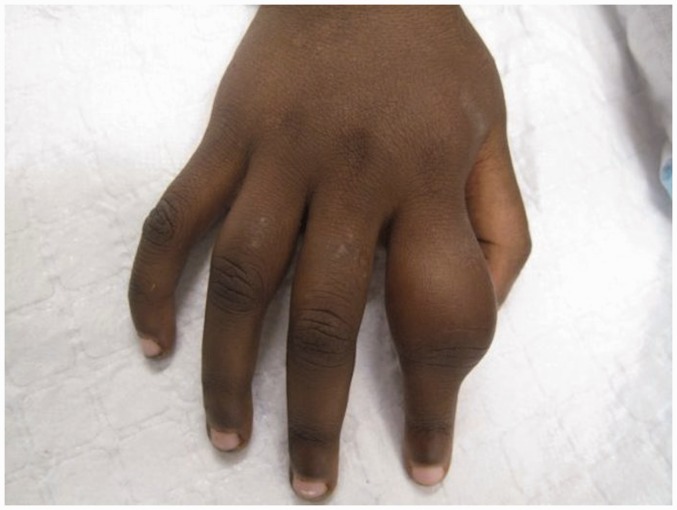
Secondary yaws: dactylitis. Copyright Oriol Mitjà.

**Figure 7.**
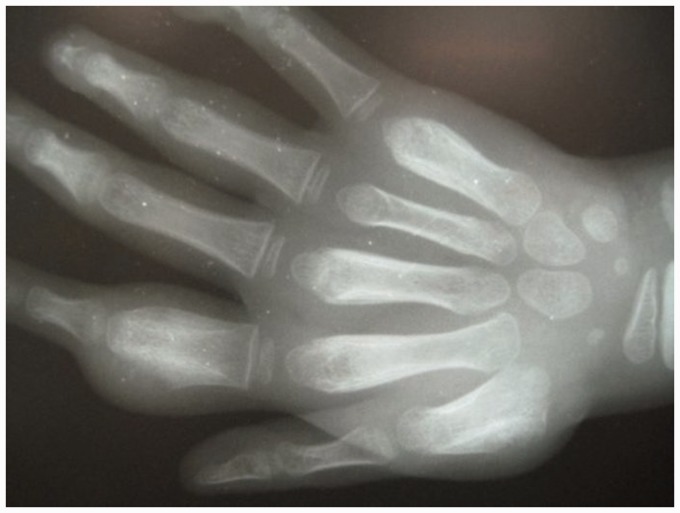
Secondary yaws: radiographic evidence of osteoperiostitis. Copyright Oriol Mitjà.

Secondary yaws typically causes osteoperiostitis of multiple bones. Involvement of long bones may cause nocturnal pain and visible periosteal thickening (Figures 6 and 7). Involvement of the proximal phalanges of the fingers manifests as polydactylitis. This contrasts with late yaws in which mono-dactylitis is typical. One study from Papua New Guinea^14^ reported joint pains in 75% of children with secondary yaws.

## Latent yaws

Individuals with latent yaws have reactive serological tests but no clinical signs. It is not known how many patients are infected without developing clinical disease. Patients with primary and secondary yaws may pass into a period of latency after resolution of clinical signs. As in syphilis, infectious relapses can occur, most commonly up to five years (rarely up to 10 years) after infection.^1,20^ Relapsing lesions tend to occur around the axillae, anus and mouth.

## Tertiary yaws

Tertiary yaws is thought to occur in about 10% of untreated patients, although its manifestations are rare in the modern era. The skin is most commonly affected. Hyperkeratosis of palms and soles and plaques may occur. Nodules may form near joints and ulcerate, causing tissue necrosis.^4^ ‘Sabre tibia’ results from chronic osteo-periostitis. *Gangosa* or rhinopharyngitis mutilans denotes mutilating facial ulceration of the palate and nasopharynx secondary to osteitis. *Goundou* was a rare complication even when yaws was hyperendemic and is characterised by exostoses of the maxillary bones.^21^

## Cardiovascular yaws

Although the consensus is that yaws does not cause cardiovascular disease, this view has been challenged. Post-mortem studies have found evidence of aortitis in patients with yaws.^22^ Histologically these lesions are similar to those found in tertiary syphilis. Despite these studies, definitive evidence of cardiovascular disease in yaws is lacking.

## Neurological yaws

The consensus that yaws does not cause neurological disease^1^ has also been challenged by studies that found neuro-ophthalmic^23^ and CSF abnormalities^24^ in patients with yaws. As with cardiovascular disease definitive evidence for a causal role of yaws in neurological disease remains absent.

## Yaws and pregnancy

While there is no laboratory evidence that *T pallidum ssp. pertenue* can cause congenital yaws, there are anecdotal reports.^11^ Most were published when serodiagnosis relied on non-treponemal tests and before treponemal IgM testing of neonates was feasible.^19^

## Yaws and HIV

There are no published data on the interaction between HIV and yaws. It is possible that patients with latent yaws might develop relapsing disease with increasing immune damage.^25^ There are also no data on the impact on other STIs, although given the low rates of genital lesions and that the disease predominantly occurs in children it might be anticipated that any effect would be minimal.

## Diagnosis

### Syphilis or yaws?

Physicians working in endemic areas usually make a presumptive diagnosis of yaws based on clinical and epidemiological features, with or without confirmatory blood tests. However, because syphilis and yaws co-exist in many tropical regions, and serology cannot distinguish between treponemal sub-species, it may be impossible to identify with certainty the causative organism. There are reports of yaws presenting in non-endemic countries.^26,27^

## Laboratory diagnosis

### Dark ground microscopy

Spirochaetes were first observed in yaws ulcers in 1905,^28^ the year in which *T pallidum ssp. pallidum* was identified in a lymph gland of a patient with syphilis. *T pallidum ssp. pertenue* is morphologically identical to *T pallidum ssp. pallidum.* As *T pallidum spp.* are only 0.3 µm wide and 6–20 µm in length, dark ground microscopy is required for visualisation. Samples from primary and secondary yaws lesions are obtained as described for syphilis.

### Polymerase chain reaction

Polymerase chain reaction (PCR) testing of samples can identify *T pallidum* but current PCR protocols do not distinguish between sub-species.^14,29^ *T pallidum ssp. pertenue* has been identified to sub-species level using real-time PCR and DNA sequencing in a child from Congo with a pruritic skin eruption,^27^ but few clinicians have access to such techniques.

### Serology

While serological tests are the bedrock of yaws diagnosis they cannot distinguish between sub-species of *T pallidum.*^30^

### Non-treponemal (cardiolipin) tests

The venereal disease research laboratory (VDRL) and rapid plasma reagin (RPR) tests use an antigen of cardiolipin, lecithin and cholesterol. Patient-derived antibodies produced against lipid in the cell surface of *T pallidum* react with antigen to cause visible flocculation. The VDRL is read microscopically whereas the RPR can be read with the naked eye. Although non-specific, VDRL/RPR titres best reflect disease activity. Titres fall after treatment and may become zero, especially after treatment of early infection.^31^ RPR titres are generally higher in primary than secondary yaws.^1^

### Treponemal tests

These include the *T pallidum* haemagglutination (TPHA) and the *T pallidum* particle agglutination (TPPA) tests. They are more specific than cardiolipin tests and usually remain positive after treatment.

Point-of-care tests have proved useful in syphilis and results of an initial study in Papua New Guinea suggest they may also be of value in the diagnosis of yaws with good sensitivity and specificity.^32^ Further studies of these tests in yaws are in progress.

## Histology

In early yaws there is marked epidermal hyperplasia and papillomatosis, often with focal spongiosis.^33^ Neutrophils accumulate in the epidermis, causing microabscesses. A dense dermal infiltrate of plasma cells is seen.^34^ In contrast with syphilis, there is little endothelial cell proliferation or vascular obliteration.^34^ *T pallidum* can be identified in tissue sections using Warthin-Starry or Levaditi silver stains. While *T pallidum ssp. pertenue* is found mainly in the epidermis, *T pallidum ssp. pallidum* is identified more in the dermis.^35^ Direct and indirect immunofluorescence and immunoperoxidase tests using specific polyclonal antibodies to *T pallidum* can also be used with histology specimens.^36^

## Radiology

Bone involvement may be revealed by radiographs even when clinical signs are absent (Figure 7).^37^

## Treatment

Benzathine penicillin-G has been the mainstay of treatment for yaws for over 60 years. Lower doses are used compared to syphilis with a recommended dose of 0.6 MU for children (under 10) and 1.2 MU for older children and adults. In a recent single-centre randomised controlled trial, one dose of azithromycin 30 mg/kg was shown to be equivalent to penicillin in patients with primary and secondary yaws, with a cure rate of approximately 95%.^38^ No other treatment strategies are supported by randomised controlled trials although data from case series suggest oral penicillin can be successful.^9^

Based on these findings, azithromycin is now central to the WHO eradication plan for yaws, which aims to employ community mass treatment in endemic regions. WHO plan to have no further cases of active yaws worldwide by 2017 and to confirm eradication by 2020.^39,40^ Despite this optimism there are several barriers to a successful eradication programme including a lack of accurate epidemiological data from many countries where yaws is reported, the absence of dedicated funding for eradication efforts and a concern that resistance to azithromycin, well described in syphilis,^41^ will emerge in yaws. Monitoring for this during the eradication programme will be essential. This ambitious plan will require considerable input from NGOs, academic institutions and policy makers.

### Response to treatment

**Figure 8.**
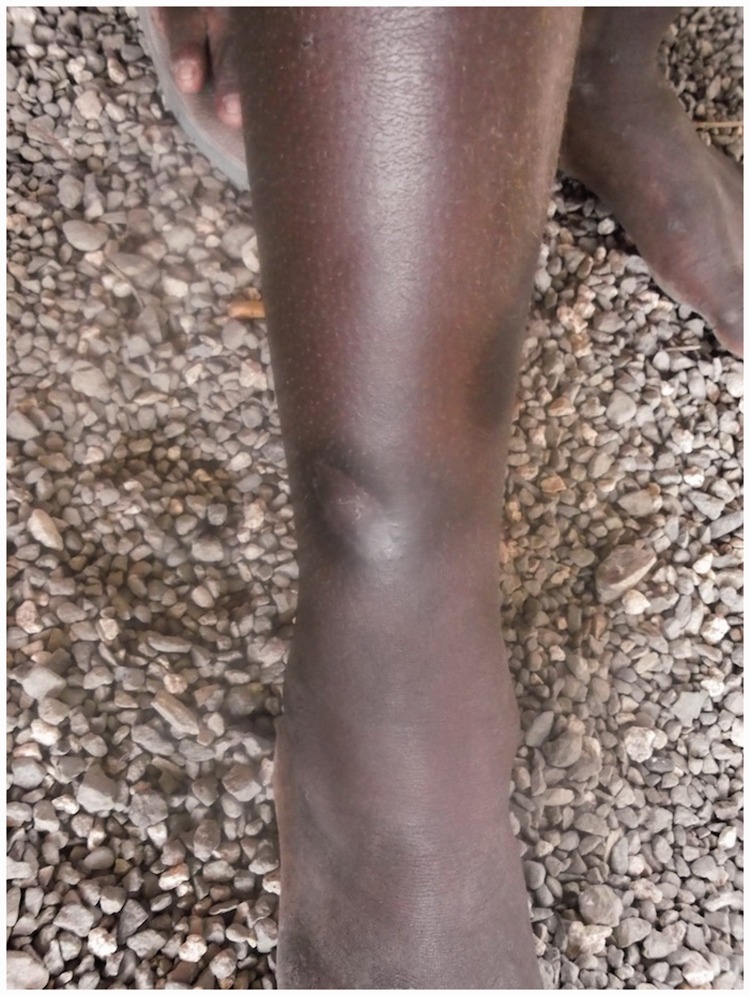
Primary yaws: healed Lesion. Copyright Michael Marks.

Treponemes disappear from lesions within 8–10 hours of treatment with penicillin. Skin lesions begin to heal within 2–4 weeks (Figure 8). In patients with secondary yaws, joint pains may begin to improve within as little as 48 hours.^42^ Bone changes are reversible if treated early enough. Following successful treatment the RPR declines and at 12 months up to 90% of individuals have either a four-fold reduction in RPR or become seronegative.^43^ Failure of skin lesions to heal or the RPR to drop should be considered treatment failure and an indication for repeat treatment. In endemic settings treatment failure is more common in individuals from higher prevalence communities.^44^ Whether this represents true treatment failure or re-infection is unclear.

The authors of a study in Papua New Guinea reported failure of yaws treatment with penicillin, which they attributed to bacterial resistance, although no laboratory evidence of this was available.^45^

## Conclusions

Yaws is still endemic in a number of countries worldwide despite a significant reduction in the number of affected individuals following mass treatment campaigns in the middle of the twentieth century. Clinicians need to be aware of the epidemiology and manifestations of yaws, which should be considered in the differential diagnosis of patients with reactive serology from endemic countries. Older individuals may have acquired yaws in countries that are no longer endemic. Routine testing cannot distinguish between syphilis and yaws. Treatment strategies are similar for the two diseases, although a lower dose of penicillin is used in yaws. Given the limitations in distinguishing the two diagnoses clinicians should consider treating for venereal syphilis in patients with reactive serology without a clear history of yaws. In this context it is important that the clinician carefully explains to the patient and their partner that reactive serology alone is not diagnostic of a sexually-transmitted route of infection.

Development of near-patient and laboratory tests specific for treponemal sub-species is long overdue. We also need to know if yaws can be transmitted from mother to child in utero and whether it can produce neurological and/or cardiovascular complications. Given the prevalence of macrolide and azalide resistance reported in *T pallidum ssp. pallidum*, it is important that surveillance of treatment efficacy is maintained in planned yaws mass treatment campaigns.
